# Transmembrane Collagens in Neuromuscular Development and Disorders

**DOI:** 10.3389/fnmol.2020.635375

**Published:** 2021-01-18

**Authors:** Tomoko Wakabayashi

**Affiliations:** Department of Innovative Dementia Prevention, Graduate School of Medicine, The University of Tokyo, Tokyo, Japan

**Keywords:** collagen, extracellular matrix, neuromuscular junction (NMJ), motor neuron, axon guidance, myasthenia gravis

## Abstract

Neuromuscular development is a multistep process and involves interactions among various extracellular and transmembrane molecules that facilitate the precise targeting of motor axons to synaptogenic regions of the target muscle. Collagenous proteins with transmembrane domains have recently emerged as molecules that play essential roles in multiple aspects of neuromuscular formation. Membrane-associated collagens with interrupted triple helices (MACITs) are classified as an unconventional subtype of the collagen superfamily and have been implicated in cell adhesion in a variety of tissues, including the neuromuscular system. Collagen XXV, the latest member of the MACITs, plays an essential role in motor axon growth within the developing muscle. In humans, loss-of-function mutations of collagen XXV result in developmental ocular motor disorders. In contrast, collagen XIII contributes to the formation and maintenance of neuromuscular junctions (NMJs), and disruption of its function leads to the congenital myasthenic syndrome. Transmembrane collagens are conserved not only in mammals but also in organisms such as *C. elegans*, where a single MACIT, COL-99, has been documented to function in motor innervation. Furthermore, in *C. elegans*, a collagen-like transmembrane protein, UNC-122, is implicated in the structural and functional integrity of the NMJ. This review article summarizes recent advances in understanding the roles of transmembrane collagens and underlying molecular mechanisms in multiple aspects of neuromuscular development and disorders.

## Introduction

For the acquisition of proper motor function, spinal motor neurons and their target muscles need to be precisely interconnected by specialized synapses called neuromuscular junctions (NMJs; Sanes and Lichtman, 1999; Bonanomi and Pfaff, 2010). Motor axons are guided to the target muscles that are simultaneously formed, and extend and branch over the myotubes toward the endplate region where pre-formed clusters of acetylcholine receptors (AChRs) are present. Axon terminals then contact the AChR clusters and nerve-derived agrin binds to the Lrp4-MuSK complex on muscle (Kim et al., 2008). This facilitates the maturation of AChR clusters in the postsynaptic region, resulting in the assembly and refinement of NMJs. Previous studies have unveiled a variety of molecules that regulate these processes, including transmembrane/secretory guidance cues and synaptic components (Bonanomi and Pfaff, 2010; Wu et al., 2010). Among them, atypical collagens with transmembrane domains have recently emerged as molecules that play essential roles in multiple aspects of neuromuscular formation.

Collagens are the most abundant proteins in the mammalian body, playing a wide range of biological roles in tissue scaffolding, cell differentiation, adhesion, migration, and tissue repair (Myllyharju and Kivirikko, 2004). Most collagens are secreted and assembled into fibrils or supramolecular structures that act as components of the extracellular matrix (ECM). However, like cell surface receptors or adhesion molecules, transmembrane collagens are expressed on the cellular membrane. Collagens XIII and XXV, the main focus of this review article, are expressed in developing muscles and play crucial roles in continuous but independent developmental processes from motor innervation to NMJ formation.

## Transmembrane Collagens

**Figure 1:**
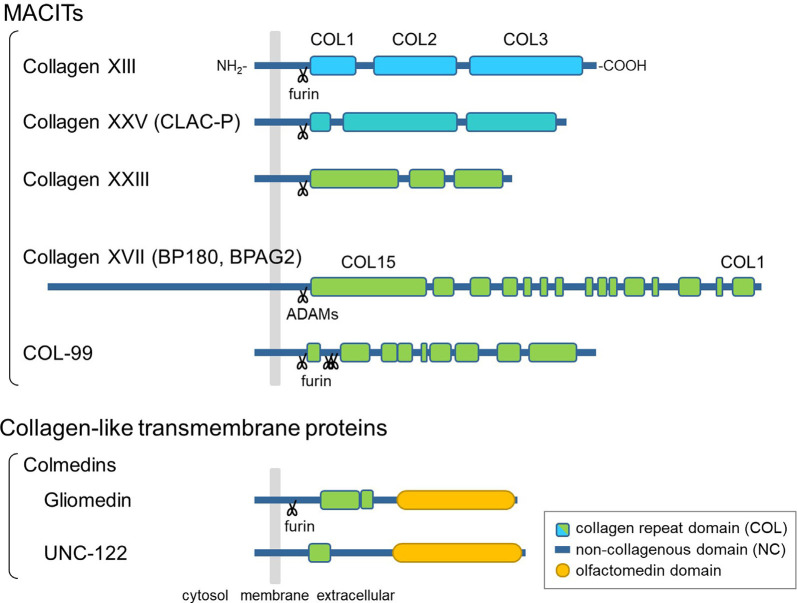
Schematic presentation of the domain structures of membrane-associated collagens with interrupted triple helices (MACITs) and collagen-like transmembrane proteins. The MACITs family collagens are shown at the top. Collagens XIII, XXV, XXIII, and XVII are the mammalian MACITs, and COL-99 is the nematode MACIT. Two collagen-like transmembrane proteins, the colmedin family proteins gliomedin and UNC-122, are shown at the bottom. The cellular membrane is shown in gray, collagen repeat domains (COL) in green, non-collagenous domains (NC) in dark blue, olfactmedin domains in yellow. Protease cleavage sites are depicted as scissors.

The hallmark of collagens is a triple-helical structure composed of polypeptides called α-chains, which contain Gly-X-Y repeat sequences (Myllyharju and Kivirikko, 2004). Among the 28 vertebrate collagen types (I–XXVIII), collagens XIII, XVII, XXIII, and XXV are members of a subfamily named membrane-associated collagens with interrupted triple helices (MACITs; Shoulders and Raines, 2009; Ricard-Blum, 2011). MACITs are type II transmembrane proteins consisting of an N-terminal cytoplasmic domain, a transmembrane domain, and a C-terminal large ectodomain (Giudice et al., 1992; Hägg et al., 1998; Hashimoto et al., 2002; Banyard et al., 2003; Figure 1). The ectodomain is composed of several collagenous (COL) domains flanked by non-collagenous (NC) domains. Once α-chains polymerize *via* the coiled-coil domains, which are important for inter-chain interactions, the triple-helix formation of MACIT proceeds in the N- to C-terminal direction, opposite to that of other, secreted collagen types (Snellman et al., 2000; Areida et al., 2001; Latvanlehto et al., 2003; McAlinden et al., 2003). Another characteristic feature of MACITs is that they can exist in two different forms: a membrane-tethered form and a shed form. Collagens XIII, XXIII, and XXV share a similar molecular structure and are cleaved by furin-like proprotein convertases at the recognition sequence immediately N-terminal to the first COL domain (Snellman et al., 2000; Hashimoto et al., 2002; Banyard et al., 2003). They are evolutionally conserved as a single gene in Ecdysozoa, such as *col-99* in *C. elegans*, and the furin cleavage sites and C-terminal sequences are highly conserved among orthologs and paralogs (Tu et al., 2015). Collagen XVII is structurally different from the other three and is cleaved by ADAM family proteases (Franzke et al., 2002, 2004).

In addition to MACITs, several collagen-like transmembrane proteins not defined as members of the collagen superfamily have been identified, such as ectodysplasin A, the colmedins, and members of the class A scavenger receptors (Ezer et al., 1999; Loria et al., 2004; Kelley et al., 2014). These proteins are all characterized as type II membrane proteins harboring one or two collagenous domain(s) and some other functional protein motifs. Like MACITs, ectodomains of ectodysplasin A and colmedins are secreted (Elomaa et al., 2001; Loria et al., 2004; Maertens et al., 2007).

Members of the MACIT family are expressed in a variety of tissues and cells. Collagen XVII, also known as BP180 or BPAG2, was the first MACIT to have its biological function elucidated (Tsuruta et al., 2011). It is a structural component of hemidesmosomes, which facilitate the adhesion of basal keratinocytes to the underlying basement membrane. Moreover, collagen XVII has been associated with congenital and acquired blistering diseases. These findings indicate that collagen XVII plays an important role in the skin as a cell adhesion molecule. The other three MACITs are structurally related and are therefore presumed to have similar functions. Of these, the physiological function of collagen XXIII has not yet been characterized. Meanwhile, recent studies on collagens XIII and XXV in genetically modified mice and in rare genetic disorders have revealed that the transmembrane collagens are important for the development of the neuromuscular system, which will be discussed in the following sections.

## Roles of Transmembrane Collagens in Neuromuscular Development in Animal Models

### Collagen XXV in Intramuscular Motor Innervation

Collagen XXV was originally identified as CLAC-P, which is a precursor of a collagenous Alzheimer amyloid plaque component, CLAC (Hashimoto et al., 2002; Söderberg et al., 2003). It is expressed exclusively in neurons in adult mammals (Hashimoto et al., 2002; Monavarfeshani et al., 2017). The ectodomain binds to amyloid-β fibrils, resulting in co-deposition with amyloid plaques in the brains of Alzheimer’s patients or model mice (Kowa et al., 2004; Osada et al., 2005; Söderberg et al., 2005; Hashimoto et al., 2020).

During embryonic development, however, collagen XXV is expressed in both neural and muscular tissues (Tanaka et al., 2014; Gonçalves et al., 2019). The biological role was revealed by a study in mice deficient in *Col25a1*, which displayed characteristics of neuromuscular defects (Tanaka et al., 2014). In *Col25a1*^−/−^ embryos, motor nerves fail to enter and branch within the target muscle. The lack of motor innervation leads to excessive apoptosis of spinal motor neurons during development, resulting in neonatal death due to respiratory failure. These abnormalities were fully reproduced by conditional disruption of *Col25a1* in developing muscles but not in motor neurons (Munezane et al., 2019), indicating an essential role for muscle-derived collagen XXV in intramuscular motor innervation.

**Figure 2:**
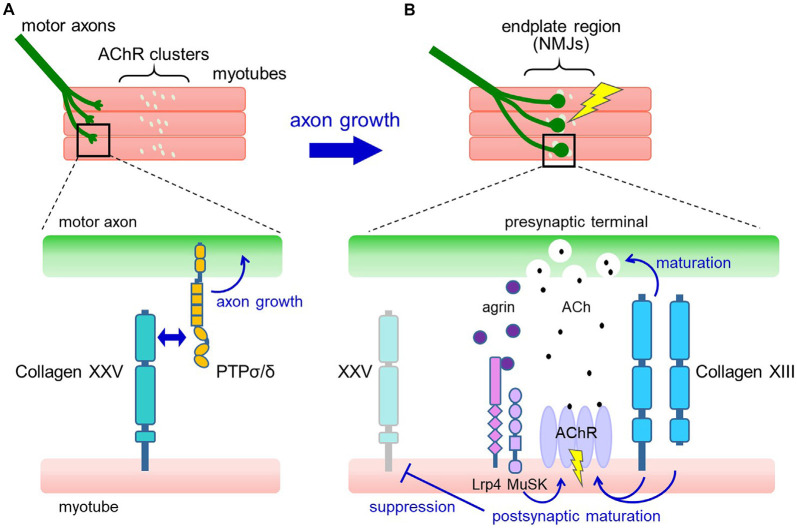
Proposed model for the roles of collagens XXV and XIII in neuromuscular development. **(A)** In developing muscle, motor axons extend and branch over the myotubes toward the region where pre-formed acetylcholine receptor (AChR) clusters are present (upper panel). Collagen XXV expressed in myotubes interacts with PTPσ and PTPδ on motor axons to induce intramuscular axon growth (lower panel). **(B)** Axon terminals contact the AChR clusters, resulting in the formation of functional neuromuscular junctions (NMJs; upper panel). At the NMJ (lower panel), neuron-derived agrin induces postsynaptic maturation through Lrp4/MuSK. Also, collagen XIII expressed in myotubes promotes both presynaptic and postsynaptic maturation. Acetylcholine (ACh) released from the presynaptic nerve terminal binds to AChRs in the synaptic region and depolarizes the myotubes, resulting in the suppression of collagen XXV expression.

The expression of *Col25a1* in muscles is transiently upregulated when myoblasts fuse to form multinucleated myotubes, and rapidly declines after the formation of NMJs. A possible mechanism for this dynamic regulation is the persistent suppression of *Col25a1* expression by signals downstream of the nerve-induced excitation of skeletal muscle (Munezane et al., 2019; Figures 2A,B). In addition, muscle-specific microRNA, miR-499, has been suggested to downregulate the expression of *Col25a1* as myotubes mature (Gonçalves et al., 2019).

How does collagen XXV in muscles induce motor innervation? *In vitro* and cell-based assays have shown that collagen XXV attracts motor axons through its interaction with PTPσ and PTPδ (Munezane et al., 2019; Figure 2A). PTPs are type IIa receptor protein tyrosine phosphatases implicated in axon elongation and regeneration as well as in synapse formation (Takahashi and Craig, 2013; Ohtake et al., 2018). Notably, the presence of an uncharacterized ligand for PTPσ expressed in developing myotubes has been suggested (Sajnani-Perez et al., 2003). Furthermore, mice doubly deficient in PTPσ and PTPδ are the only models that phenocopy the severe developmental deficits observed in *Col25a1* knockouts, supporting the functional link between collagen XXV and PTPs (Uetani et al., 2006).

Previous studies of neuromuscular development have shown that the lack of essential components involved in NMJ formation or pharmacological blockade of action potentials in developing muscles leads to axon overgrowth far beyond the AChR clusters. This predicted the presence of muscle-derived signals that retrogradely promote intramuscular axon growth, which can be suppressed by muscle excitation (Dahm and Landmesser, 1988; DeChiara et al., 1996). The interaction between collagen XXV and PTPσ/δ may satisfy the features of this long-predicted signaling, and future validation of its sufficiency and elucidation of the downstream molecular pathways are expected.

### Collagen XIII in NMJ Maturation

Collagen XIII is distributed at the sites of cell-cell and cell-matrix interactions in a variety of developing and adult tissues such as skeletal muscle, heart, and neural structures, suggesting that it is involved in cell adhesion (Peltonen et al., 1999; Sandberg-lall et al., 2000; Hägg et al., 2001; Sund et al., 2001). Particularly in skeletal muscle, collagen XIII is rich in the AChR-positive postsynaptic regions on the motor endplate and myotendinous junction (Hägg et al., 2001; Latvanlehto et al., 2010).

Analyses of mice lacking collagen XIII (*Col13a1^−/−^*) revealed a role for collagen XIII in the formation of NMJs (Latvanlehto et al., 2010). Although *Col13a1*^−/−^ mice are viable, they showed delayed/incomplete AChR cluster development, aberrant NMJ morphology, and compromised synaptic function. Furthermore, transgenic overexpression of collagen XIII under the mouse endogenous *Col13a1* promoter induced mislocalization of collagen XIII in the extrasynaptic regions of skeletal muscle, resulting in abnormal NMJ maturation (Härönen et al., 2019). Thus, proper expression of collagen XIII in skeletal muscle is essential for the formation and function of NMJs.

Mechanistically, collagen XIII has been suggested to regulate both presynaptic and postsynaptic maturation (Figure 2B). In *Col13a1^−/−^* mice, synaptic vesicles failed to accumulate properly in presynaptic terminals (Latvanlehto et al., 2010). In contrast, *Col13a1*^tm/tm^ mice that solely express cleavage-deficient collagen XIII had fully aligned synaptic vesicles and a rather elevated number of presynaptic active zones (Härönen et al., 2017). Thus, the transmembrane form of collagen XIII retrogradely induces presynaptic differentiation of the NMJ, presumably through trans-synaptic adhesion. In terms of postsynaptic maturation, the collagen XIII ectodomain promotes the development of postsynaptic AChR clusters from immature plaque-like to mature pretzel-like structures in C2C12 myotubes in an autocrine manner (Latvanlehto et al., 2010). *In vivo*, the study of *Col13a1*^tm/tm^ mice suggests that the presence of membrane-tethered collagen XIII is sufficient for postsynaptic maturation (Härönen et al., 2017). Collagen XIII binds to ColQ, the collagenous tail of acetylcholinesterase (AChE), and regulates the distribution of AChE. This may partly explain the mechanisms that promote postsynaptic maturation (Härönen et al., 2017). These observations collectively imply a dominant role for full-length collagen XIII in NMJ maturation and a homeostatic role for the shed ectodomain.

In addition to NMJ formation, collagen XIII has also been shown to play a role in the regeneration process of NMJs. In *Col13a1^−/−^* mice but not in *Col13a1*^tm/tm^ mice, regeneration and functional recovery following nerve crush were severely impaired and incomplete, indicating the importance of membrane-tethered collagen XIII in NMJ recovery after peripheral nerve injury (Zainul et al., 2018).

### Nematode MACIT, COL-99

A genetic screen revealed that COL-99 is involved in axon guidance (Taylor et al., 2018). COL-99 is the only ortholog of collagens XIII, XXIII, and XXV in *C. elegans* and has a similar domain structure: a transmembrane domain, multiple collagenous domains, and putative furin cleavage sites in the juxtamembrane domain (Tu et al., 2015; Figure 1). Mutations in the *col-99* gene lead to defects in the axonal projection of the major longitudinal tracts, e.g., the ventral nerve cord (VNC) and dorsal nerve cord (DNC; Taylor et al., 2018). The affected nerve cords are formed by axons of several classes of neurons, including motor neurons. COL-99 is expressed in the hypodermis during development and navigates axon outgrowth longitudinally, possibly through the discoidin domain receptors DDR-1 and DDR-2, receptor tyrosine kinases expressed by neurons. Given that COL-99 is also localized in adult muscles, especially in the NMJs, the MACITs might have conserved functions in the neuromuscular system of a wide range of species (Tu et al., 2015; Taylor et al., 2018).

### Collagen-Like Transmembrane Proteins

Colmedins are a family of type II membrane proteins harboring collagenous and olfactomedin domains in the extracellular region (Loria et al., 2004; Tomarev and Nakaya, 2009; Figure 1). Gliomedin, a mammalian colmedin, is widely expressed in neural tissues and regulates the formation of the nodes of Ranvier by serving as a glial ligand for the axonal adhesion molecules NrCAM and neurofascin (Eshed et al., 2005; Maertens et al., 2007). In contrast, a *C. elegans* colmedin UNC-122 is expressed in muscles and is localized in the postsynaptic sites of the NMJs (Loria et al., 2004). *unc-122* mutant worms displayed neurotransmission defects and therefore caused an uncoordinated (unc) phenotype. Although the functional domains and interacting molecules of UNC-122 remain unclear, it has been postulated that postsynaptically localized UNC-122 is involved in creating a microenvironment required for the structural and functional integrity of the NMJs in *C. elegans*.

## Neuromuscular Disorders

### Loss-of-Function of Collagen XXV Leads to Cranial Nerve Dysinnervation

The cranial nerves are 12 pairs of nerves that arise directly from the brain, nine of which contain a motor component. A heterogeneous group of congenital syndromes characterized by abnormalities in the development and wiring of cranial nerves has been collectively referred to as congenital cranial dysinnervation disorders (CCDDs; Gutowski et al., 2003). A growing number of causative genes for CCDDs have been identified, which are associated with syndromes affecting the movement of the eye, eyelid, and facial muscles, e.g., congenital fibrosis of the extraocular muscles (CFEOM), Duane syndrome, and Möbius syndrome (Gutowski and Chilton, 2015). Evidence obtained from genetic, clinical, and biological studies suggests that CCDDs primarily result from neurodevelopmental abnormalities (Oystreck et al., 2011).

Recently, a novel subtype of CCDD associated with *COL25A1* was reported as CFEOM5 (OMIM #616219; Shinwari et al., 2015). Patients with CCDD related to *COL25A1* display disturbances of ocular motility, e.g., congenital ptosis and exotropic Duane syndrome, suggesting abnormal innervation of the extraocular muscles (Khan and Al-Mesfer, 2015). Consistent with the clinical findings, systemic or muscle-specific loss of murine *Col25a1* resulted in a significant reduction in the number of motor neurons in the cranial nerve nuclei, including the oculomotor, trochlear, trigeminal, and facial motor nuclei at E18.5. Accordingly, abnormalities in motor innervation of muscles of the head, such as the extraocular and masseter muscles, were observed (Munezane et al., 2019). Notably, *COL25A1* is the first gene among the causative genes of CCDD, whose expression in muscles may account for the pathomechanism.

Genetic analyses have identified a homozygous missense mutation in the COL2 domain (p.Gly382Arg) and a compound heterozygous mutation (p.Gly497Ter and a 12.4 kb deletion spanning exons 4–10; Shinwari et al., 2015). Another variant of *COL25A1* has also been reported in an exome analysis of families with CCDD-like phenotypes (Akawi et al., 2015). Biochemical and cellular studies revealed that the CCDD mutations in *COL25A1* severely impaired the interaction of collagen XXV with PTPσ/δ, thereby reducing the ability to attract motor axons (Munezane et al., 2019). Thus, the collagen XXV-PTPσ/δ interaction might also be essential for the development of cranial motor neurons, including those innervating the extraocular muscles.

### Collagen XIII Is Associated With Neuromuscular Junction Disorders

Congenital myasthenic syndromes (CMS) are a group of inherited neuromuscular disorders caused by abnormal synaptic transmission. Although over 30 causative genes have been reported, which encode proteins that function in postsynaptic, presynaptic, or basal lamina compartments, there are still CMS cases left with unidentified genetic mutations (Vanhaesebrouck and Beeson, 2019). Over the past years, next-generation sequencing analyses have revealed that mutations in *COL13A1* cause autosomal recessive CMS, designated as CMS19 (OMIM #616720). These include nonsense, frameshift, splice-site, and missense mutations, which are scattered throughout the gene (Logan et al., 2015; Dusl et al., 2019; Marquardt and Li, 2019; Rodríguez Cruz et al., 2019). Mutations leading to premature termination could trigger nonsense-mediated mRNA decay or produce C-terminally truncated proteins that might lack functional domains (Logan et al., 2015). All the missense mutations affect evolutionarily conserved amino acid residues, including glycine residues in the first position of the Gly-X-Y repeat sequence (Rodríguez Cruz et al., 2019). The functional effect of the CMS mutation was examined by introducing the *COL13A1* c.1171delG frameshift mutation in C2C12 myoblasts. The mutation reduced the number of agrin-induced AChR clusters on differentiated myotubes, indicating the deleterious effects of CMS mutation on postsynaptic maturation (Logan et al., 2015). The muscles of CMS19 patients showed abnormalities in neuromuscular transmission on electrophysiological analysis, and histological analysis also showed mild changes such as variations in fiber size, the presence of some inner nuclei, and disturbed distribution of AChE (Logan et al., 2015; Rodríguez Cruz et al., 2019).

The clinical features of CMS due to *COL13A1* are early-onset muscle weakness with feeding and breathing difficulties. The pattern of muscle weakness includes facial and axial weakness as well as ptosis with limited fatigability. The disease course is variable, but CMS19 causes severe symptoms early in life and gradually improves in some cases (Rodríguez Cruz et al., 2019). Such clinical features may be explained by the results of animal studies in which collagen XIII plays a major role in the process of NMJ formation. The combination of β2-adrenergic receptor agonist salbutamol and potassium channel blocker 3,4-diaminopyridine was effective in improving motor and respiratory function in CMS19 patients (Logan et al., 2015; Dusl et al., 2019; Rodríguez Cruz et al., 2019). Salbutamol and 3,4-diaminopyridine have been suggested to enhance synaptic integrity and ACh release, respectively, and may therefore compensate for the functional loss of collagen XIII.

Collagen XIII may also be an autoantigen for myasthenia gravis (MG). MG is a disease caused by autoantibodies targeting components of the motor endplate, resulting in muscle weakness and fatigability. Although the majority of patients possess antibodies against AChR, an increasing number of other antigenic targets have been identified (Gilhus et al., 2016). In a study using sera from MG patients, collagen XIII autoantibodies were detected in 5 of 70 patients tested, and three of them were AChR antibody seronegative (Tu et al., 2018). No discernible symptomatic differences were observed among MG patients with and without collagen XIII autoantibodies. Further analyses are needed to clarify the specificity of collagen XIII for MG and its therapeutic or diagnostic value.

## Conclusion

Findings from experimental and pathophysiological studies highlight the cell adhesion properties of transmembrane collagens in neuromuscular development. These collagens have markedly different structural features from traditional adhesion molecules; in the case of collagen XIII, the approximately 150 nm long ectodomain is rod-shaped with hinges that correspond to the NC domains (Tu et al., 2002). These considerably large and flexible molecular properties might be important for its function in connecting gaps in the neuromuscular synaptic cleft of about 50–100 nm or between myotubes and incoming axons loosely surrounded by Schwann cell precursors. Furthermore, by the nature of collagenous domains, collagens XIII and XXV have been reported to interact with multiple ECM components such as fibronectin, nidogen, and glycosaminoglycan chains (Tu et al., 2002; Osada et al., 2005). Thus, the shed ectodomain, which is a characteristic of transmembrane collagens, may not only relieve the function of full-length molecules but may also exert unique roles in the neuromuscular ECM.

The functions indicated by animal studies mostly explain the disease mechanisms affected by mutations in collagens XIII and XXV. However, phenotypic and histopathological changes were more severe in the gene-deficient mice than in humans. Particularly for collagen XXV, the reason that the mutational effects are limited to impairments in the EOMs in humans remains unsolved. Possible explanations might be that partial loss of function due to mutations only affects vulnerable muscle types, or that neuron-muscle interactions in humans involve a certain contribution of other molecules, including the closely related transmembrane collagens. Elucidating the molecular basis of the bioactive and pathological mechanisms of transmembrane collagens in the future will be of fundamental importance in neurodevelopmental research. Given the regulatory function of collagen XIII in the structures of NMJs and the axon-inducing effects of collagen XXV, transmembrane collagens may also have the ability to facilitate the regenerative process from nerve and muscle damage and degeneration caused by neuromuscular disorders; collagen XIII has indeed been implicated in neuromuscular synapse regeneration (Zainul et al., 2018). From this perspective, future research on transmembrane collagens may also lead to clinical applications in various neuromuscular disorders.

## Author Contributions

TW wrote the manuscript and created the figures.

## Conflict of Interest

The author declares that the research was conducted in the absence of any commercial or financial relationships that could be construed as a potential conflict of interest.

## References

[B1] Akawi N. McRae J. Ansari M. Balasubramanian M. Blyth M. Brady A. F.. (2015). Discovery of four recessive developmental disorders using probabilistic genotype and phenotype matching among 4,125 families. Nat. Genet. 47, 1363–1369. 10.1038/ng.3410 26437029 PMC5988033

[B2] Areida S. K. Reinhardt D. P. Müller P. K. Fietzek P. P. Köwitz J. Marinkovich M. P.. (2001). Properties of the collagen type XVII ectodomain. Evidence for n- to c-terminal triple helix folding. J. Biol. Chem. 276, 1594–1601. 10.1074/jbc.M008709200 11042218

[B3] Banyard J. Bao L. Zetter B. R. (2003). Type XXIII collagen, a new transmembrane collagen identified in metastatic tumor cells. J. Biol. Chem. 278, 20989–20994. 10.1074/jbc.M210616200 12644459

[B4] Bonanomi D. Pfaff S. L. (2010). Motor axon pathfinding. Cold Spring Harb. Perspect. Biol. 2:a001735. 10.1101/cshperspect.a001735 20300210 PMC2829954

[B5] Dahm L. M. Landmesser L. T. (1988). The regulation of intramuscular nerve branching during normal development and following activity blockade. Dev. Biol. 130, 621–644. 10.1016/0012-1606(88)90357-0 3058544

[B6] DeChiara T. M. Bowen D. C. Valenzuela D. M. Simmons M. V. Poueymirou W. T. Thomas S.. (1996). The receptor tyrosine kinase MuSK is required for neuromuscular junction formation *in vivo*. Cell 85, 501–512. 10.1016/s0092-8674(00)81251-9 8653786

[B7] Dusl M. Moreno T. Munell F. Macaya A. Gratacòs M. Abicht A.. (2019). Congenital myasthenic syndrome caused by novel COL13A1 mutations. J. Neurol. 266, 1107–1112. 10.1007/s00415-019-09239-7 30767057

[B8] Elomaa O. Pulkkinen K. Hannelius U. Mikkola M. Saarialho-Kere U. Kere J. (2001). Ectodysplasin is released by proteolytic shedding and binds to the EDAR protein. Hum. Mol. Genet. 10, 953–962. 10.1093/hmg/10.9.953 11309369

[B9] Eshed Y. Feinberg K. Poliak S. Sabanay H. Sarig-Nadir O. Spiegel I.. (2005). Gliomedin mediates schwann cell-axon interaction and the molecular assembly of the nodes of ranvier. Neuron 47, 215–229. 10.1016/j.neuron.2005.06.026 16039564

[B10] Ezer S. Bayés M. OutiElomaa O. Schlessinger D. Kere J. (1999). Ectodysplasin is a collagenous trimeric type II membrane protein with a tumor necrosis factor-like domain and co-localizes with cytoskeletal structures at lateral and apical surfaces of cells. Hum. Mol. Genet. 8, 2079–2086. 10.1093/hmg/8.11.2079 10484778

[B11] Franzke C.-W. Tasanen K. Borradori L. Huotari V. Bruckner-Tuderman L. (2004). Shedding of collagen XVII/BP180 structural motifs influence cleavage from cell surface. J. Biol. Chem. 279, 24521–24529. 10.1074/jbc.M308835200 15047704

[B12] Franzke C.-W. Tasanen K. Schäcke H. Zhou Z. Tryggvason K. Mauch C.. (2002). Transmembrane collagen XVII, an epithelial adhesion protein, is shed from the cell surface by ADAMs. EMBO J. 21, 5026–5035. 10.1093/emboj/cdf532 12356719 PMC129053

[B13] Gilhus N. E. Skeie G. O. Romi F. Lazaridis K. Zisimopoulou P. Tzartos S. (2016). Myasthenia gravis—autoantibody characteristics and their implications for therapy. Nat. Rev. Neurol. 12, 259–268. 10.1038/nrneurol.2016.44 27103470

[B14] Giudice G. J. Emery D. J. Diaz L. A. (1992). Cloning and primary structural analysis of the bullous pemphigoid autoantigen BP180. J. Invest. Dermatol. 99, 243–250. 10.1111/1523-1747.ep12616580 1324962

[B15] Gonçalves T. J. M. Boutillon F. Lefebvre S. Goffin V. Iwatsubo T. Wakabayashi T.. (2019). Collagen XXV promotes myoblast fusion during myogenic differentiation and muscle formation. Sci. Rep. 9:5878. 10.3892/ol.2020.12332 30971718 PMC6458142

[B17] Gutowski N. J. Bosley T. M. Engle E. C. (2003). 110th ENMc international workshop: the congenital cranial dysinnervation disorders (CCDDs): naarden, The Netherlands, 25–27 October, 2002. Neuromuscul. Disord. 13, 573–578. 10.1016/s0960-8966(03)00043-9 12921795

[B16] Gutowski N. J. Chilton J. K. (2015). The congenital cranial dysinnervation disorders. Arch. Dis. Child. 100, 678–681. 10.1136/archdischild-2014-307035 25633065

[B18] Hägg P. Rehn M. Huhtala P. Väisänen T. Tamminen M. Pihlajaniemi T. (1998). Type XIII collagen is identified as a plasma membrane protein. J. Biol. Chem. 273, 15590–15597. 10.1074/jbc.273.25.15590 9624150

[B19] Hägg P. Väisänen T. Tuomisto A. Rehn M. Tu H. Huhtala P.. (2001). Type XIII collagen: a novel cell adhesion component present in a range of cell-matrix adhesions and in the intercalated discs between cardiac muscle cells. Matrix Biol. 19, 727–742. 10.1016/s0945-053x(00)00119-0 11223332

[B20] Härönen H. Zainul Z. Naumenko N. Sormunen R. Miinalainen I. Shakirzyanova A.. (2019). Correct expression and localization of collagen XIII are crucial for the normal formation and function of the neuromuscular system. Eur. J. Neurosci. 49, 1491–1511. 10.1111/ejn.14346 30667565

[B21] Härönen H. Zainul Z. Tu H. Naumenko N. Sormunen R. Miinalainen I.. (2017). Collagen XIII secures pre- and postsynaptic integrity of the neuromuscular synapse. Hum. Mol. Genet. 26, 2076–2090. 10.1093/hmg/ddx101 28369367

[B22] Hashimoto T. Fujii D. Naka Y. Kashiwagi-Hakozaki M. Matsuo Y. Matsuura Y.. (2020). Collagenous Alzheimer amyloid plaque component impacts on the compaction of amyloid-β plaques. Acta Neuropathol. Commun. 8:212. 10.1186/s40478-020-01075-5 33287899 PMC7720522

[B23] Hashimoto T. Wakabayashi T. Watanabe A. Kowa H. Hosoda R. Nakamura A.. (2002). CLAC: a novel Alzheimer amyloid plaque component derived from a transmembrane precursor, CLAC-P/collagen type XXV. EMBO J. 21, 1524–1534. 10.1093/emboj/21.7.1524 11927537 PMC125364

[B24] Kelley J. L. Ozment T. R. Li C. Schweitzer J. B. Williams D. L. (2014). Scavenger receptor-A (CD204): a two-edged sword in health and disease. Crit. Rev. Immunol. 34, 241–261. 10.1615/critrevimmunol.2014010267 24941076 PMC4191651

[B25] Khan A. O. Al-Mesfer S. (2015). Recessive COL25A1 mutations cause isolated congenital ptosis or exotropic Duane syndrome with synergistic divergence. J. AAPOS 19, 463–465. 10.1016/j.jaapos.2015.04.011 26486031

[B26] Kim N. Stiegler A. L. Cameron T. O. Hallock P. T. Gomez A. M. Huang J. H.. (2008). Lrp4 is a receptor for agrin and forms a complex with MuSK. Cell 135, 334–342. 10.1016/j.cell.2008.10.002 18848351 PMC2933840

[B27] Kowa H. Sakakura T. Matsuura Y. Wakabayashi T. Mann D. M. A. Duff K.. (2004). Mostly separate distributions of CLAC- versus Aβ40- or thioflavin S-reactivities in senile plaques reveal two distinct subpopulations of β-amyloid deposits. Am. J. Pathol. 165, 273–281. 10.1016/s0002-9440(10)63295-6 15215182 PMC1618534

[B28] Latvanlehto A. Fox M. A. Sormunen R. Tu H. Oikarainen T. Koski A.. (2010). Muscle-derived collagen XIII regulates maturation of the skeletal neuromuscular junction. J. Neurosci. 30, 12230–12241. 10.1523/JNEUROSCI.5518-09.2010 20844119 PMC2955218

[B29] Latvanlehto A. Snellman A. Tu H. Pihlajaniemi T. (2003). Type XIII collagen and some other transmembrane collagens contain two separate coiled-coil motifs, which may function as independent oligomerization domains. J. Biol. Chem. 278, 37590–37599. 10.1074/jbc.M305974200 12832406

[B30] Logan C. V. Cossins J. Rodríguez Cruz P. M. Parry D. A. Maxwell S. Martínez-Martínez P.. (2015). Congenital myasthenic syndrome type 19 is caused by mutations in COL13A1, encoding the atypical non-fibrillar collagen type XIII α1 chain. Am. J. Hum. Genet. 97, 878–885. 10.1016/j.ajhg.2015.10.017 26626625 PMC4678414

[B31] Loria P. M. Hodgkin J. Hobert O. (2004). A conserved postsynaptic transmembrane protein affecting neuromuscular signaling in *caenorhabditis elegans*. J. Neurosci. 24, 2191–2201. 10.1523/JNEUROSCI.5462-03.2004 14999070 PMC6730426

[B32] Maertens B. Hopkins D. Franzke C.-W. Keene D. R. Bruckner-Tuderman L. Greenspan D. S.. (2007). Cleavage and oligomerization of gliomedin, a transmembrane collagen required for node of ranvier formation. J. Biol. Chem. 282, 10647–10659. 10.1074/jbc.M611339200 17293346

[B33] Marquardt R. J. Li Y. (2019). Congenital myasthenic syndrome type 19 due to a novel mutation in the COL13A1 GENE. Muscle Nerve 60, E3–E4. 10.1002/mus.26494 31018245

[B34] McAlinden A. Smith T. A. Sandell L. J. Ficheux D. Parry D. A. D. Hulmes D. J. S. (2003). α-helical coiled-coil oligomerization domains are almost ubiquitous in the collagen superfamily. J. Biol. Chem. 278, 42200–42207. 10.1074/jbc.M302429200 12920133

[B35] Monavarfeshani A. Knill C. N. Sabbagh U. Su J. Fox M. A. (2017). Region- and cell-specific expression of transmembrane collagens in mouse brain. Front. Integr. Neurosci. 11:20. 10.3389/fnint.2017.00020 28912695 PMC5583603

[B36] Munezane H. Oizumi H. Wakabayashi T. Nishio S. Hirasawa T. Sato T.. (2019). Roles of collagen XXV and its putative receptors PTPσ/δ in intramuscular motor innervation and congenital cranial dysinnervation disorder. Cell Rep. 29, 4362.e6–4376.e6. 10.1016/j.celrep.2019.11.112 31875546

[B37] Myllyharju J. Kivirikko K. I. (2004). Collagens, modifying enzymes and their mutations in humans, flies and worms. Trends Genet. 20, 33–43. 10.1016/j.tig.2003.11.004 14698617

[B38] Ohtake Y. Saito A. Li S. (2018). Diverse functions of protein tyrosine phosphatase σ in the nervous and immune systems. Exp. Neurol. 302, 196–204. 10.1016/j.expneurol.2018.01.014 29374568 PMC6275553

[B39] Osada Y. Hashimoto T. Nishimura A. Matsuo Y. Wakabayashi T. Iwatsubo T. (2005). CLAC binds to amyloid β peptides through the positively charged amino acid cluster within the collagenous domain 1 and inhibits formation of amyloid fibrils. J. Biol. Chem. 280, 8596–8605. 10.1074/jbc.M413340200 15615705

[B40] Oystreck D. T. Engle E. C. Bosley T. M. (2011). Recent progress in understanding congenital cranial dysinnervation disorders. J. Neuroophthalmol. 31, 69–77. 10.1097/WNO.0b013e31820d0756 21317732 PMC3524829

[B41] Peltonen S. Hentula M. Hägg P. Ylä-Outinen H. Tuukkanen J. Lakkakorpi J.. (1999). A novel component of epidermal cell-matrix and cell-cell contacts: transmembrane protein type XIII collagen. J. Invest. Dermatol. 113, 635–642. 10.1046/j.1523-1747.1999.00736.x 10504453

[B42] Ricard-Blum S. (2011). The collagen family. Cold Spring Harb. Perspect. Biol. 3:a004978. 10.1101/cshperspect.a004978 21421911 PMC3003457

[B43] Rodríguez Cruz P. M. Cossins J. de Paula Estephan E. Munell F. Selby K. Hirano M.. (2019). The clinical spectrum of the congenital myasthenic syndrome resulting from COL13A1 mutations. Brain 142, 1547–1560. 10.1093/brain/awz107 31081514 PMC6752227

[B44] Sajnani-Perez G. Chilton J. K. Aricescu A. R. Haj F. Stoker A. W. (2003). Isoform-specific binding of the tyrosine phosphatase PTPσ to a ligand in developing muscle. Mol. Cell. Neurosci. 22, 37–48. 10.1016/s1044-7431(02)00026-x 12595237

[B45] Sandberg-lall M. Hägg P. O. Wahlström I. Pihlajaniemi T. (2000). Type XIII collagen is widely expressed in the adult and developing human eye and accentuated in the ciliary muscle, the optic nerve and the neural retina. Exp. Eye Res. 70, 401–410. 10.1006/exer.1998.0826 10865988

[B46] Sanes J. R. Lichtman J. W. (1999). Development of the vertebrate neuromuscular junction. Annu. Rev. Neurosci. 22, 389–442. 10.1146/annurev.neuro.22.1.389 10202544

[B47] Shinwari J. M. A. Khan A. Awad S. Shinwari Z. Alaiya A. Alanazi M.. (2015). Recessive mutations in COL25A1 are a cause of congenital cranial dysinnervation disorder. Am. J. Hum. Genet. 96, 147–152. 10.1016/j.ajhg.2014.11.006 25500261 PMC4289688

[B48] Shoulders M. D. Raines R. T. (2009). Collagen structure and stability. Annu. Rev. Biochem. 78, 929–958. 10.1146/annurev.biochem.77.032207.120833 19344236 PMC2846778

[B49] Snellman A. Tu H. Väisänen T. Kvist A.-P. Huhtala P. Pihlajaniemi T. (2000). A short sequence in the N-terminal region is required for the trimerization of type XIII collagen and is conserved in other collagenous transmembrane proteins. EMBO J. 19, 5051–5059. 10.1093/emboj/19.19.5051 11013208 PMC302104

[B50] Söderberg L. Kakuyama H. Möller A. Ito A. Winblad B. Tjernberg L. O.. (2005). Characterization of the Alzheimer’s disease-associated CLAC protein and identification of an amyloid β-peptide-binding site. J. Biol. Chem. 280, 1007–1015. 10.1074/jbc.M403628200 15522881

[B51] Söderberg L. Zhukareva V. Bogdanovic N. Hashimoto T. Winblad B. Iwatsubo T.. (2003). Molecular identification of AMY, an Alzheimer disease amyloid-associated protein. J. Neuropathol. Exp. Neurol. 62, 1108–1117. 10.1093/jnen/62.11.1108 14656069

[B52] Sund M. Väisänen T. Kaukinen S. Ilves M. Tu H. Autio-Harmainen H.. (2001). Distinct expression of type XIII collagen in neuronal structures and other tissues during mouse development. Matrix Biol. 20, 215–231. 10.1016/s0945-053x(01)00134-2 11470398

[B53] Takahashi H. Craig A. M. (2013). Protein tyrosine phosphatases PTPδ, PTPσ, and LAR: presynaptic hubs for synapse organization. Trends Neurosci. 36, 522–534. 10.1016/j.tins.2013.06.002 23835198 PMC3789601

[B54] Tanaka T. Wakabayashi T. Oizumi H. Nishio S. Sato T. Harada A.. (2014). CLAC-P/collagen Type XXV is required for the intramuscular innervation of motoneurons during neuromuscular development. J. Neurosci. 34, 1370–1379. 10.1523/JNEUROSCI.2440-13.2014 24453327 PMC6705307

[B55] Taylor J. Unsoeld T. Hutter H. (2018). The transmembrane collagen COL-99 guides longitudinally extending axons in *C. elegans*. Mol. Cell. Neurosci. 89, 9–19. 10.1016/j.mcn.2018.03.003 29550247

[B56] Tomarev S. I. Nakaya N. (2009). Olfactomedin domain-containing proteins: possible mechanisms of action and functions in normal development and pathology. Mol. Neurobiol. 40, 122–138. 10.1007/s12035-009-8076-x 19554483 PMC2936706

[B57] Tsuruta D. Hashimoto T. Hamill K. J. Jones J. C. R. (2011). Hemidesmosomes and focal contact proteins: functions and cross-talk in keratinocytes, bullous diseases and wound healing. J. Dermatol. Sci. 62, 1–7. 10.1016/j.jdermsci.2011.01.005 21376539 PMC4492441

[B58] Tu H. Huhtala P. Lee H.-M. Adams J. C. Pihlajaniemi T. (2015). Membrane-associated collagens with interrupted triple-helices (MACITs): evolution from a bilaterian common ancestor and functional conservation in *C. elegans*. BMC Evol. Biol. 15:281. 10.1186/s12862-015-0554-3 26667623 PMC4678570

[B59] Tu H. Pirskanen-Matell R. Heikkinen A. Oikarainen T. Risteli J. Pihlajaniemi T. (2018). Autoimmune antibodies to collagen XIII in myasthenia gravis patients. Muscle Nerve 57, 506–510. 10.1002/mus.25969 28885698

[B60] Tu H. Sasaki T. Snellman A. Göhring W. Pirilä P. Timpl R.. (2002). The type XIII collagen ectodomain is a 150-nm rod and capable of binding to fibronectin, nidogen-2, perlecan, and heparin. J. Biol. Chem. 277, 23092–23099. 10.1074/jbc.M107583200 11956183

[B61] Uetani N. Chagnon M. J. Kennedy T. E. Iwakura Y. Tremblay M. L. (2006). Mammalian motoneuron axon targeting requires receptor protein tyrosine phosphatases σ and δ. J. Neurosci. 26, 5872–5880. 10.1523/JNEUROSCI.0386-06.2006 16738228 PMC6675220

[B62] Vanhaesebrouck A. E. Beeson D. (2019). The congenital myasthenic syndromes: expanding genetic and phenotypic spectrums and refining treatment strategies. Curr. Opin. Neurol. 32, 696–703. 10.1097/WCO.0000000000000736 31361628 PMC6735524

[B63] Wu H. Xiong W. C. Mei L. (2010). To build a synapse: signaling pathways in neuromuscular junction assembly. Development 137, 1017–1033. 10.1242/dev.038711 20215342 PMC2835321

[B64] Zainul Z. Heikkinen A. Koivisto H. Rautalahti I. Kallio M. Lin S.. (2018). Collagen XIII is required for neuromuscular synapse regeneration and functional recovery after peripheral nerve injury. J. Neurosci. 38, 4243–4258. 10.1523/JNEUROSCI.3119-17.2018 29626165 PMC6596032

